# Supplementary material for: Storage Fungi and Mycotoxins Associated with Rice Samples Commercialized in Thailand
Source: Foods. 2023 Jan 20;12(3):487. doi: 10.3390/foods12030487 (PMC9914209; doi:10.3390/foods12030487)
Supplement: Supplementary file 1 [file foods-12-00487-s001.zip › foods-2129076-supplementary.pdf]

Table S1. *Aspergillus*, *Penicillium*, *Talaromyces* and related genera used in phylogenetic analyses and their GenBank accession numbers.

Taxon	Original strain number	NCBI GenBank accession number			
		ITS	*BenA*	*CaM*	*RPB2*
A. aculeatus	ATHUM 5028	EU982028	EU982087	N/A	N/A
A. aeneus.	NRRL 4769	EF652474	EF652298	N/A	EF652210
A. arenarius	NRRL 5012	EU021615	EU021674	N/A	EU021653
A. awamori	ATHUM 5181	EU982010	EU982069	N/A	EU982094
A. bisporus.	NRRL 3693	EF661208	EF661121	N/A	EF661077
A. brunneouniseriatus	NRRL 4273	EU982028	EF652123	N/A	EF652089
A. campestris	NRRL 13001	EF652474	EF669577	N/A	EF669619
A. clavatoflavus	NRRL 5113	EU021615	EF669686	N/A	EF669668
A. clavatus	ATHUM 5032	EU982010	EU982073	N/A	EU982098
A. clavatus	ATHUM 5036	EF661208	EU982074	N/A	EU982099
A. elongatus	NRRL 5176	EF652502	EF652326	N/A	EF652238
A. flavus	ATHUM 5015	EU982011	EU982070	N/A	EU982095
A. flavus	ATHUM 5033	EU982012	EU982071	N/A	EU982096
A. fumigatus	ATHUM 5013	EU982013	EU982072	N/A	EU982097
A. giganteus	NRRL 10	EF669928	EF669789	N/A	EF669716
A. janus	NRRL 1787	EU021598	EU014076	N/A	EF669620
A. niger	ATHUM 2539	EU982009	EU982068	N/A	EU982092
A. niger	ATHUM 5044	EU982008	EU982067	N/A	EU982093
A. niveus	ATHUM 5029	EU982023	EU982082	N/A	EU982105
A. ochraceus	ATHUM 4958	EU982029	EU982088	N/A	EU982111
A. oryzae	ATHUM 4958	EU982022	EU982081	N/A	EU982104
A. oryzae	ATHUM 5037	EU982020	EU982079	N/A	N/A
A. parasiticus	ATHUM 5038	EU982021	EU982080	N/A	N/A
A. puniceus	ATHUM 5434	EU982019	EU982078	N/A	EU982101
A. restrictus	NRRL 154	EF652042	EF651880	N/A	EF651978
A. sclerotiorum	NRRL 415	EF661400	EF661337	N/A	EF661287
A. sydowii	ATHUM 5093	EU982025	N/A	N/A	N/A
A. terreus	ATHUM 4761	EU982024	EU982083	N/A	EU982106
A. terreus	ATHUM 5097	EU982026	EU982085	N/A	EU982108
A. ustus	ATHUM 5103	EU982027	EU982086	N/A	EU982109
A. versicolor	ATHUM 2541	EU982032	EU982091	N/A	EU982114
Emericella nidulans	ATHUM 5022	EU982031	EU982090	N/A	EU982113
E. variecolor	ATHUM 5019	EU982030	EU982089	N/A	EU982112
Eurotium amstelodami	ATHUM 5082	EU982017	EU982076	N/A	N/A
E. rubrum	ATHUM 5183	EU982018	EU982077	N/A	N/A
Neosartorya fischeri	ATHUM 5030	EU982016	EU982075	N/A	EU982100
Metarhizium anisopliae	-	AF218207	AY995134	N/A	DQ522453
A. niger	CBS 554.65^T	AJ223852	AY585536	AJ964872	N/A
A. tubingensis	CBS 134.48^T	AJ223853	AY820007	AJ964876	N/A
A. oryzae	NRRL 447	EF661560	EF661483	EF661506	EF661438
Talaromyces islandicus	CBS 338.48^T	NR103664	KF984655	KF984655	JN121495
T. pinophilus	CBS 631.66^T	JN899382	JX091381	KF741964	KM023291
T. radicus	CBS 100489^T	JN899324	KF984599	KF984773	KF985013
P. citrinum	CBS 139.45^T	MH856132	GU944545	N/A	JF417416

Taxon	Original strain number	NCBI GenBank accession number			
		ITS	*BenA*	*CaM*	*RPB2*
P. chermesinum	CBS 231.81^T	AY742693	KJ834441	N/A	JN406581
P. vietnamense	VTCC 930029	MT102836	MT230561	ON209438	MT222288
P. indicum	CBS 115.63^T	AY742699	EU427263	AY741744	JN406640
P. coffeae	CBS 119387^T	AY742702	KJ834443	AY741747	JN121436
T. purpureogenum	CBS 286.36^T	JN899372	N/A	JX315655	JX315709
T. indigoticus	CBS 100534^T	NR137076	JX494308	KF741931	N/A
T. macrosporus	CBS 317.63^T	NR145155	JX091382	KF741952	KM023292
T. wortmannii	CBS 391.48^T	KF984829	KF984648	KF984756	JN121669
T. tardifaciens	CBS 250.94^T	KF984874	KF984560	KF984682	KF984908
T. unicus	CBS 100535^T	NR_157429	KJ865735	KJ885283	MN969150
A. niger	**B3-01**	OP946385	OP965320	OP980962	OP965302
A. niger	**B8-02**	OP946386	OP938582	OP980963	OP965303
A. niger	**B9-03**	OP946387	OP965321	OP980964	OP965304
A. niger	**B16-04**	OP946388	OP965322	OP980965	OP965305
A. niger	**G17-01**	OP946389	OP965323	OP980966	OP965306
A. niger	**K6-03**	OP946390	OP965324	OP980967	OP965307
A. niger	**K10-02**	OP946391	OP938583	OP980968	OP965308
A. niger	**M5-03**	OP946392	OP938584	OP980969	OP965317
***Aspergillus* sp.**	**B1-01**	OP946393	OP965325	OP980970	OP965309
***Aspergillus* sp.**	**G11-01**	OP946394	OP938587	OP980971	OP965310
***Penicillium* sp.**	**G11-02**	OP946395	OP965326	N/A	OP965318
***Penicillium* sp.**	**G11-05**	OP946396	OP938588	OP980972	OP965311
***Talaromyces* sp.**	**B8-03**	OP946397	OP965327	N/A	N/A
***Talaromyces* sp.**	**B5-02**	OP946398	N/A	OP980973	OP965312
***Talaromyces* sp.**	**K1-02**	OP946399	N/A	OP980974	OP965313
***Talaromyces* sp.**	**M1-01**	OP946400	OP965328	OP980975	OP965314
***Talaromyces* sp.**	**M15-04**	OP946401	OP965329	OP980976	OP965315
***Talaromyces* sp.**	**W2-03**	OP946402	OP965330	OP980977	OP965319
***Talaromyces* sp.**	**W3-04**	OP946403	OP938585	OP980978	OP965316

New sequences and tentative names in this study based on gross morphological identification are in bold. N/A = not available; T = Ex-type strain. ATHUM: Culture Collection of Fungi, National and Kapodistrian University of Athens; NRRL: National Center for Agricultural Utilization Research; CBS, culture collection of the CBS-KNAW Fungal Biodiversity Centre, Utrecht, Netherlands; VTCC: Vietnam Type Culture Collection.

Table S2. The BLAST tool (NCBI) and a tentative fungal taxon (ID) based on morphological and sequence-based data were used to determine the sequence identity (%) of closely related species.

Original Code	Morphological identification	BLAST search against the strains in this study compared with the closest relatives and GenBank accession number in NCBI based on the ITS, *BenA*, *CaM*, and *RPB2* sequences using BLAST program												Proposed fungal taxon (ID) based on sequence-based data and phylogenetic tree (Figure 3)
		The closest taxon based on ITS sequence			The closest taxon based on *BenA* sequence			The closest taxon based on *CaM* sequence			The closest taxon based on *RPB2* sequence			
		Putative taxonomic affinity and GenBank accession No.		Identity (%)	Putative taxonomic affinity and GenBank accession No.		Identity (%)	Putative taxonomic affinity and GenBank accession No.		Identity (%)	Putative taxonomic affinity and GenBank accession No.		Identity (%)	
B1-01	*Aspergillus* sp.	*A. fumigatus*	ON033650	100	*A. fumigatus*	KF669422	100	*A. fumigatus*	MG991470	100	*A. fumigatus*	MT384369	99.43	*A. fumigatus*
B3-01	*A. niger*	*Aspergillus* sp.	KX928746	99.83	*A. niger*	OL689145	99.82	*A. niger*	MG991511	100	*A. niger*	LN833566	100	*A. niger*
B8-02	*A. niger*	*Aspergillus* sp	KT374059	99.84	*A. niger*	OL689145	99.82	*A. niger*	MK451457	99.84	*A. niger*	LN833566	99.66	
B9-03	*A. niger*	*A. niger*	MH752206	99.67	*A. awamori*	MH447369	99.82	*A. niger*	GU195633	100	*A. niger*	LN833566	99.42	
B16-04	*A. niger*	*Aspergillus* sp.	KR822140	99.83	*A. niger*	OL689145	100	*A. niger*	MK451457	100	*A. welwitschiae*	MK450818	100	
G17-01	*A. niger*	*A. niger*	MH752206	99.67	*A. niger*	KC175288	99.81	*A. niger*	MH614646	100	*A. niger*	MT318291	99.89	
K6-03	*A. niger*	*A. niger*	KF305756	99.67	*A. niger*	KC175288	99.81	*A. niger*	MH614646	100	*A. niger*	EU982092	100	
K10-02	*A. niger*	*Aspergillus* sp.	KT374059	99.58	*A. niger*	OL689145	100	*A. niger*	KX894628	99.69	*A. welwitschiae*	MK450818	99.89	
M5-03	*A. niger*	*A. niger*	MG991581	99.83	*A. niger*	OL689145	100	*A. niger*	KJ599596	99.72	*A. niger*	KT824060	99.58	
G11-01	*Aspergillus* sp.	*A. tubingensis*	MG991653	99.83	*A. tubingensis*	HQ632719	99.82	*A. costaricensis*	MH644993	99.59	*A. neoniger*	MK450786	100	*A. tubingensis*
B5-02	*Penicillium* sp./ *Talaromyces* sp.	*Talaromyces radicus*	ON394881	100	*Paramyrothecium foenicuchlicola*	KU846409	84.52	*T.radicus*	KY053252	100	*T.radicus*	KF196971	100	*T. radicus*
B8-03	*Penicillium* sp./ *Talaromyces* sp.	*T. purpureogenum*	KC344977	100	*T. purpureogenum*	KC345006	99.15	ND	ND	ND	ND	ND	ND	*T. purpureogenum*
G11-02	*Penicillium* sp.	*P. citrinum*	MN826202	100	*P. citrinum*	KC345003	99.60	ND	ND	ND	*P. citrinum*	KJ476426	99.62	*P. citrinum*
G11-05	*Penicillium* sp.	*P. chermesinum*	MW081295	100	*P. chermesinum*	KJ767035	100	*P. chermesinum*	MT302215	100	*P. chermesinum*	MK450829	99.79	*P. chermesinum*
K1-02	*Penicillium*/ *Talaromyces* sp.	*T. islandicus*	KF031335	100	*T. islandicus*	MH644074	88.89	*T. islandicus*	KF984777	100	*T. islandicus*	KF196969	100	*T. islandicus*
M1-01	*Penicillium*/ *Talaromyces* sp.	*T. pinophilus*	CP017345	99.83	*T. pinophilus*	MH909370	100	*T. pinophilus*	MH909450	99.38	*T. pinophilus*	MH9095341	99.89	*T.pinophilus*
M15-04	*Penicillium*/ *Talaromyces* sp.	*Talaromyces* sp.	MN330064	99.83	*T. pinophilus*	MN330064	100	*T. pinophilus*	KY053247	98.97	*T. pinophilus*	MH909534	100	
W2-03	*Penicillium*/ *Talaromyces* sp.	*Penicillium* sp.	HQ631042	99.83	*T. pinophilus*	HQ631042	100	*T. pinophilus*	MH909418	99.41	*T. pinophilus*	MH909534	99.55	
W3-04	*Penicillium*/ *Talaromyces* sp.	*T. pinophilus*	MK336445	100	*T. pinophilus*	KM066141	100	*T. pinophilus*	MH909418	99.41	*T. pinophilus*	MH909534	99.89	

ND = not detected

Table S3. MS/MS parameters for the determination of 15 mycotoxins.

Analyte	Precusor ion (m/z)	Product ions (m/z)	Collision energy (eV)	Fragmentor (V)	Retention time (min)	Polarity
AFB_1	313.07	285.1	21	150	6.32	Positive
		241.0[a]	35	150		
AFB_2	315.09	287.1	25	160	6.18	Positive
		259.0[a]	29	160		
AFG_1	329.07	311.0	25	160	6.16	Positive
		243.0[a]	43	160		
AFG_2	331.08	313.0	25	180	6.02	Positive
		245.0[a]	29	180		
OTA	404.00	192.9	48	130	6.79	Positive
		102.1[a]	80	130		
ZEA	319.16	283.0	5	80	7.24	Positive
		187.0[a]	17	80		
BEA	801.40	784.3	13	160	9.13	Positive
		244.1[a]	35	160		
FB1	722.40	352.5	40	160	5.59	Positive
		334.4[a]	45	160		
FB2	706.30	336.2	35	200	5.99	Positive
		318.3[a]	40	200		
T2	489.40	387.3	20	170	7.00	Positive
		245.2[a]	26	170		
DAS	384.20	307.1	5	60	6.28	Positive
		199.0[a]	13	60		
ALT	259.10	187.9	25	240	6.08	Positive
		160.1[a]	33	240		
CIT	251.10	233.1	3	120	5.79	Positive
		205.1[a]	20	120		
DON	355.10	265.1	4	90	3.71	Negative
		59.1[a]	10	90		
NIV	371.10	281.0	4	80	2.26	Negative
		59.1[a]	10	80		

a= qualitative product ion.

Table S4. Performance characteristics of the proposed method.

Analyte	Groups of white rice			Groups of colored rice			Culture medium		
	LOD (ug/kg)	LOQ (ug/kg)	R^2	LOD (ug/kg)	LOQ (ug/kg)	R^2	LOD (ug/kg)	LOQ (ug/kg)	R^2
AFB_1	0.30	0.98	0.9965	0.35	1.17	0.9980	0.25	0.85	0.9990
AFB_2	0.21	0.71	0.9975	0.27	0.86	0.9982	0.21	0.63	0.9995
AFG_1	0.15	0.50	0.9980	0.22	0.73	0.9990	0.16	0.52	0.9993
AFG_2	0.20	0.67	0.9982	0.25	0.83	0.9955	0.17	0.55	0.9985
OTA	0.18	0.60	0.9997	0.20	0.69	0.9990	0.19	0.62	0.9995
ZEA	8.25	27.50	0.9953	12.5	41.67	0.9965	7.73	25.5	0.9980
BEA	0.20	0.68	0.9995	0.28	0.93	0.9990	0.15	0.51	0.9996
FB_1	0.31	1.04	0.9990	0.47	1.55	0.9995	0.38	1.25	0.9975
FB_2	0.44	1.48	0.9978	0.66	2.20	0.9980	0.53	1.77	0.9985
T2	0.80	2.67	0.9935	1.55	5.17	0.9950	0.98	3.26	0.9985
DAS	2.00	6.67	0.9941	2.75	9.17	0.9980	2.05	6.84	0.9992
DON	7.80	25.11	0.9935	12.5	41.67	0.9950	8.47	28.2	0.9965
NIV	25.0	83.33	0.9928	28.2	92.8	0.9945	25.6	85.3	0.9970
ALT	0.55	1.85	0.9977	0.70	2.33	0.9985	0.56	1.88	0.9991
CIT	0.65	2.17	0.9990	0.85	2.83	0.9978	0.67	2.23	0.9992

Table S5. Accuracy and precision study in groups of white rice, groups of colored rice and culture medium samples.

Mycotoxin	**Groups of white rice**									**Groups of colored rice**									**Culture medium**								
	Recovery (%) (n = 7)			Intra-day precision (%RSD) (n=7)			Inter-day precision (%RSD) (n = 21)			Recovery (%) (n = 7)			Intra-day precision (%RSD) (n = 7)			Inter-day precision (%RSD) (n=21)			Recovery (%) (n = 7)			Intra-day precision (%RSD) (n = 7)			Inter-day precision (%RSD) (n=21)		
	L1	L2	L3	L1	L2	L3	L1	L2	L3	L1	L2	L3	L1	L2	L3	L1	L2	L3	L1	L2	L3	L1	L2	L3	L1	L2	L3
AFB_1	87.8	90.4	95.6	3.8	3.3	3.4	4.2	3.9	2.1	83.3	86.8	89.5	5.5	5.7	4.4	8.1	6.7	5.9	90.4	92.5	95.7	2.7	1.9	2.3	3.2	3.9	4.8
AFB_2	89.4	90.1	102.4	7.6	2.7	2.8	5.7	2.1	1.3	86.4	88.1	88.6	4.9	4.5	4.6	8.5	7.6	5.4	88.1	92.6	93.3	3.4	2.2	2.4	3.8	5.8	4.5
AFG_1	90.6	92.5	98.6	7.7	5.7	2.3	6.1	2.7	2.5	85.2	87.5	93.4	5.2	4.4	5.0	6.2	9.1	5.8	92.5	94.5	88.2	2.5	2.6	3.0	3.4	2.7	3.6
AFG_2	86.8	85.7	98.4	6.5	2.7	2.4	3.5	1.8	3.9	88.1	88.4	90.3	6.1	5.9	4.7	5.1	7.2	7.1	89.6	88.7	86.4	4.5	2.5	2.5	2.8	2.3	2.7
OTA	92.7	94.1	101.5	3.2	5.4	3.0	3.6	2.4	5.8	92.8	90.4	91.7	3.8	4.3	2.9	8.1	7.2	4.2	93.3	90.2	97.6	3.2	3.7	4.6	3.0	2.5	5.2
ZEA	85.6	89.2	88.6	5.2	2.2	2.5	4.2	3.4	6.4	83.4	88.3	87.9	6.7	6.4	7.5	9.1	8.4	7.5	88.7	89.4	90.5	4.0	3.8	2.7	5.2	3.7	4.5
BEA	95.5	95.7	99.2	4.9	1.7	1.8	4.6	2.8	3.2	89.7	91.8	93.4	5.3	4.1	3.6	9.3	4.9	5.0	98.4	100.6	103.7	4.6	2.3	2.3	4.9	2.8	3.3
FB_1	90.3	93.3	93.5	7.4	1.6	4.4	5.5	2.3	2.2	79.5	83.7	85.6	4.5	4.9	5.1	4.8	8.1	5.9	90.3	92.5	89.4	5.4	2.8	2.7	5.9	5.1	3.1
FB_2	89.5	91.7	92.2	7.7	1.8	2.6	6.2	2.4	1.7	81.4	86.6	80.2	5.3	5.1	4.8	5.4	8.6	6.6	91.6	90.6	93.6	4.1	2.6	3.0	4.5	5.3	3.4
T2	88.4	91.4	95.6	5.6	2.2	2.4	5.7	3.0	6.4	81.3	84.8	88.6	4.4	3.8	3.9	7.9	7.3	3.2	90.8	88.4	92.7	4.2	2.5	2.0	5.5	6.2	2.8
DAS	89.3	94.5	97.4	4.3	2.2	2.7	4.5	2.5	5.4	85.7	87.9	90.4	3.9	4.2	4.5	5.1	5.5	4.5	89.8	90.7	91.5	4.7	4.5	4.2	5.7	4.7	4.8
DON	82.7	85.6	87.7	4.5	2.6	6.3	8.3	5.6	4.9	78.2	82.6	80.3	6.5	7.8	7.9	5.1	7.2	8.1	85.3	87.5	88.2	4.5	4.1	5.7	6.9	3.6	4.5
NIV	77.9	83.2	83.6	6.6	2.5	7.7	8.5	4.4	4.7	74.5	79.7	83.6	7.1	6.9	8.3	2.5	7.0	5.3	80.1	85.4	88.6	4.1	2.8	3.9	6.7	4.2	3.6
ALT	87.3	92.5	90.7	2.8	3.7	3.0	4.2	2.6	2.6	86.8	89.4	92.4	4.8	4.6	3.9	4.3	5.9	5.7	88.5	91.8	92.7	2.8	3.1	2.7	4.2	3.3	4.2
CIT	88.5	88.7	90.3	3.2	1.8	3.5	4.7	2.4	1.4	85.3	88.8	90.6	3.2	4.7	2.8	3.4	5.5	6.5	89.8	93.7	93.4	3.2	2.7	2.5	4.8	3.8	3.2

Note: L1 = Spiking level 1: AFB_1, AFB_2, AFG_1, AFG_2, OTA and BEA: 1 μg/kg; FB_1, FB_2 and ALT: 2 μg/kg; T2 and CIT: 5 μg/kg; DAS: 10 μg/kg; ZEA and DON: 50 μg/kg; NIV: 100 μg/kg

L2 = Spiking level 2: AFB_1, AFB_2, AFG_1, AFG_2, OTA and BEA: 10 μg/kg; FB_1, FB_2 and ALT: 20 μg/kg; T2 and CIT: 50 μg/kg; DAS: 100 μg/kg; ZEA and DON: 250 μg/kg; NIV: 500 μg/kg

L3 = Spiking level 3: AFB_1, AFB_2, AFG_1, AFG_2, OTA and BEA: 40 μg/kg; FB_1, FB_2 and ALT: 100 μg/kg; T2 and CIT: 200 μg/kg; DAS: 250 μg/kg; ZEA and DON: 500 μg/kg; NIV: 1,000 μg/kg
